# Non-invasive Imaging of Idiopathic Pulmonary Fibrosis Using Cathepsin Protease Probes

**DOI:** 10.1038/srep19755

**Published:** 2016-01-22

**Authors:** Nimali P. Withana, Xiaowei Ma, Helen M. McGuire, Martijn Verdoes, Wouter A. van der Linden, Leslie O. Ofori, Ruiping Zhang, Hao Li, Laura E. Sanman, Ke Wei, Shaobo Yao, Peilin Wu, Fang Li, Hui Huang, Zuojun Xu, Paul J. Wolters, Glenn D. Rosen, Harold R. Collard, Zhaohui Zhu, Zhen Cheng, Matthew Bogyo

**Affiliations:** 1 Department of Pathology, Stanford University School of Medicine, Stanford, CA 94305 USA; 2 Department of Radiology, Stanford University School of Medicine, Stanford, CA 94305 USA; 3 Department of Microbiology and Immunology, Stanford University School of Medicine, Stanford, CA 94305 USA; 4 Department of Chemical and Systems Biology, Stanford University School of Medicine, Stanford, CA 94305 USA; 5 Department of Pulmonary and Critical Care Medicine, Stanford University School of Medicine, Stanford, CA 94305 USA; 6 Department of Nuclear Medicine, Beijing, 100730, China; 7 Respiratory Disease, Peking Union Medical College Hospital, Chinese Academy of Medical Science & Peking Union Medical College, Beijing, 100730, China; 8 Department of Medicine, University of California San Francisco, San Francisco, CA 94143 USA

## Abstract

Idiopathic pulmonary fibrosis (IPF) is a lethal, chronic, progressive disease characterized by formation of scar tissue within the lungs. Because it is a disease of unknown etiology, it is difficult to diagnose, to predict disease course and to devise treatment strategies. Recent evidence suggests that activated macrophages play key roles in the pathology of IPF. Therefore, imaging probes that specifically recognize these pools of activated immune cells could provide valuable information about how these cells contribute to the pathobiology of the disease. Here we demonstrate that cysteine cathepsin-targeted imaging probes can be used to monitor the contribution of macrophages to fibrotic disease progression in the bleomycin-induced murine model of pulmonary fibrosis. Furthermore, we show that the probes highlight regions of macrophage involvement in fibrosis in human biopsy tissues from IPF patients. Finally, we present first-in-human results demonstrating non-invasive imaging of active cathepsins in fibrotic lesions of patients with IPF. Together, our findings validate small molecule cysteine cathepsin probes for clinical PET imaging and suggest that they have the potential to be used to generate mechanistically-informative molecular information regarding cellular drivers of IPF disease severity and progression.

Pulmonary fibrosis is a process in which fibrotic lesions form in the lung resulting in scarring and progressive morbidity (physiological restriction and impaired oxygen diffusion). Fibrotic damage is the endpoint of many disorders of the lung including the idiopathic interstitial pneumonias, chronic hypersensitivity pneumonitis, collagen vascular diseases with lung involvement, and chronic lung infections^1^. Of these conditions involving lung fibrosis, Idiopathic Pulmonary Fibrosis (IPF) is considered the archetype. IPF is a progressive and fatal lung disease of unknown cause. Current estimates of disease incidence are 40–50 per 100,000 and include approximately 125,000 cases in the United States^2^,^3^. Most patients are 50 to 70 years old, but patients with familial IPF tend to present earlier^3^,^4^,^5^. Patients are usually symptomatic for 6 to 24 months before diagnosis but often present with advanced fibrotic disease. Despite therapy, IPF has a median survival of only 4–5 years^4^,^5^. There are two recently approved therapies for IPF in the United States, but neither has been shown to stop disease progression or improve survival^6^,^7^. The lack of curative treatments is largely due to the unique pathogenesis of IPF and our lack of understanding of the factors that regulate disease course.

There is recent strong evidence to suggest that immune cells such as monocytes and macrophages play important roles in the overall disease pathology. While it is likely that repeated cycles of lung epithelial injury followed by accumulation and activation of fibroblasts in IPF are significant drivers of disease pathogenesis, recent studies suggest that activated macrophages are also likely key contributors^8^. For example, injured type II alveolar epithelial cells produce cytokines leading to accumulation of activated macrophages at fibroblastic foci^9^. This activated macrophage population also produces transforming growth factor beta (TGF-β) and CCL18, potent pro-fibrotic cytokines that are key mediators of lung fibrosis. CCL18 is produced preferentially by alternatively activated macrophages and high serum levels of this cytokine in IPF patients is associated with a higher incidence of disease progression^10^. Serum CCL18 has also been shown to predict lung disease worsening in systemic sclerosis^11^. Finally, a recent study found that surfactant protein D (SP-D) deficiency leads to macrophage infiltration and the production of the pro-fibrotic cytokines, TGF- β and platelet derived growth factor AA (PDGF-AA) in the BLM lung fibrosis model^12^.

Cysteine cathepsins are a group of proteases with elastinolytic and collagenolytic activities that are involved in various aspects of ECM remodeling^13^. Of the 11 members of this cysteine protease family, cathepsins B, L and S contribute to the degradation of several types of collagen and elastin in the extracellular space^14^. These proteases are also secreted into the extracellular space and the bronchoalveolar lavage (BAL) fluid in pulmonary disorders^15^. Furthermore, the activities of these proteases in lung tissue homogenates and activated alveolar macrophages are elevated during tissue regeneration and remodeling^16^. Because cathepsins are highly expressed in activated macrophages^17^,^18^, they are potential diagnostic and therapeutic targets in IPF disease pathogenesis.

Reagents that can detect activated forms of the cysteine cathepsins are valuable tools for highlighting sites of disease pathology involving macrophage infiltration^19^,^20^,^21^. We have developed small molecule optical imaging probes that specifically report on the activity of cysteine cathepsins^22^. These probes can be used to image populations of activated macrophages that have high expression of the cysteine cathepsins. We have also demonstrated that positron emission tomography (PET) versions of cathepsin probes can be used for non-invasive imaging of cancer margins as the result of the high content of activated macrophages within solid tumours^23^.

In this study, we utilize the bleomycin-induced murine model of lung fibrosis, tissues from human clinical samples and first-in-human imaging studies to demonstrate the value of non-invasive imaging of cysteine cathepsin activity to understand the role of these proteases in IPF disease pathobiology. Our results confirmed an increased uptake of the imaging agent in fibrotic lesions suggesting a role for activated macrophages in the pathobiology of the human disease.

## Results

### Generation of molecular probes for dual optical/PET imaging of activated macrophages

**Figure 1:**
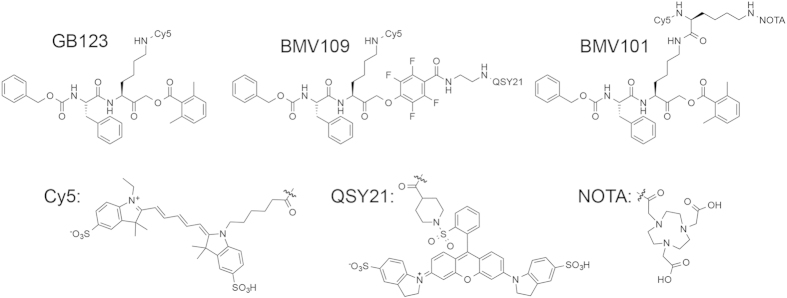
Structures of the activity based probes (ABPs) GB123, BMV109 and BMV101.

We have developed several classes of activity based probes (ABPs) targeting cysteine cathepsins^18^,^19^,^20^,^22^,^24^. In particular, the probe GB123, containing a 2,6-dimethylbenzoic acid-derived acyloxymethyl ketone (AOMK) electrophile has been validated in numerous *in vivo* optical imaging applications and was shown to target cathepsins B, S and L^19^,^25^,^26^,^27^. Recently, we demonstrated that a cathepsin ABP containing a phenoxymethyl ketone (PMK) electrophile, BMV109, has enhanced *in vivo* properties and broad reactivity towards cathepsins X, B, S and L^22^ and can be used for imaging of tumour margins *in vivo*^21^. Based on these highly successful probes, we designed a dual labelled ABP, BMV101, that could be used for both optical and PET/CT imaging applications (Fig. 1). This new probe contains a Cy5 near infrared (NIR) fluorophore for optical detection and a NOTA chelator for labelling with ^64^Cu, Al^18^F or ^68^Ga for PET/CT imaging applications. Importantly, the new probe, BMV101, performed nearly identically to BMV109 and GB123, with respect to labelling of cathepsins B, S and L in intact cells (Supplementary Fig. S1).

### Imaging disease progression in the bleomycin-induced model of lung fibrosis

**Figure 2:**
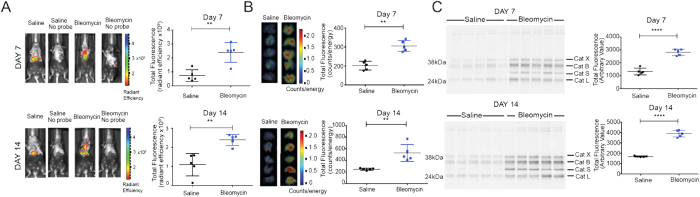
Application of the ABP BMV109 to image active cathepsins in the bleomycin mouse model of lung fibrosis. (**A**) Non-invasive optical images of mice treated with saline or bleomycin and (**B**) corresponding *ex vivo* fluorescence intensity from excised lungs for the different treatment groups. Error bars indicate mean ± SEM., n = 5 mice per group. **p < 0.01 by *t*-test. (**C**) SDS-PAGE analysis followed by flatbed laser scanning to detect probe labelled cathepsins in lung tissue lysates from mice from the different treatment groups. Lung lysate samples are for each individual mouse where n = 5 in each group. Total cathepsin labelling intensity was measured using imageJ and plotted for each group. ****p < 0.0001 by *t*-test. For completed image of uncropped gel see supplemental information files.

We first performed a study with the optimized optical probe, BMV109, to determine if cysteine cathepsins could be used as markers of pulmonary fibrosis in the lung. Bleomycin has been used extensively to model pulmonary fibrosis in rodents, with direct administration into the lungs being highly effective in eliciting fibrosis^28^. We assessed the extent of probe accumulation in the lungs, focusing on a time course between 7 and 14 days (Fig. 2A), a time period in which immune cell infiltration and initiation of fibrosis takes place^29^. Disease burden was evident in the bleomycin treated group at both time-points after bleomycin administration as measured by total body weight loss and increased lung weight (Supplementary Fig. S2A). Lung tissue at day 7 and at day 14 showed histological evidence of pulmonary fibrosis, at both time points and was greatest at day 14 (Supplementary Fig. S2B). We were also able to confirm the onset of fibrosis by increased lung collagen content at Day 14 (Supplementary Fig. S2B).

For optical imaging studies, we injected mice with the BMV109 probe via tail vein at each time-point and non-invasively quantified Cy5 fluorescence in the lung region 4 hours after probe injection (Fig. 2A). At Day 7, bleomycin-treated mice showed significantly higher fluorescence signal in the lungs when compared to the saline controls. Signal intensity further increased at Day 14. Probe signal was also detected in the liver and kidney of both groups due to clearance of the probe. We found that levels of fluorescence in the excised lungs directly correlated with the levels observed using non-invasive optical imaging methods (Fig. 2B). Subsequent analysis of lung tissues by SDS-PAGE confirmed that the primary targets of the probe in these tissues were cathepsins X, B, S and L, and that levels of labelling of these targets correlated with the extent of disease burden and intensity of signals observed by non-invasive imaging (Fig. 2C).

### Immune cell contribution to disease progression in the bleomycin lung fibrosis model

**Figure 3:**
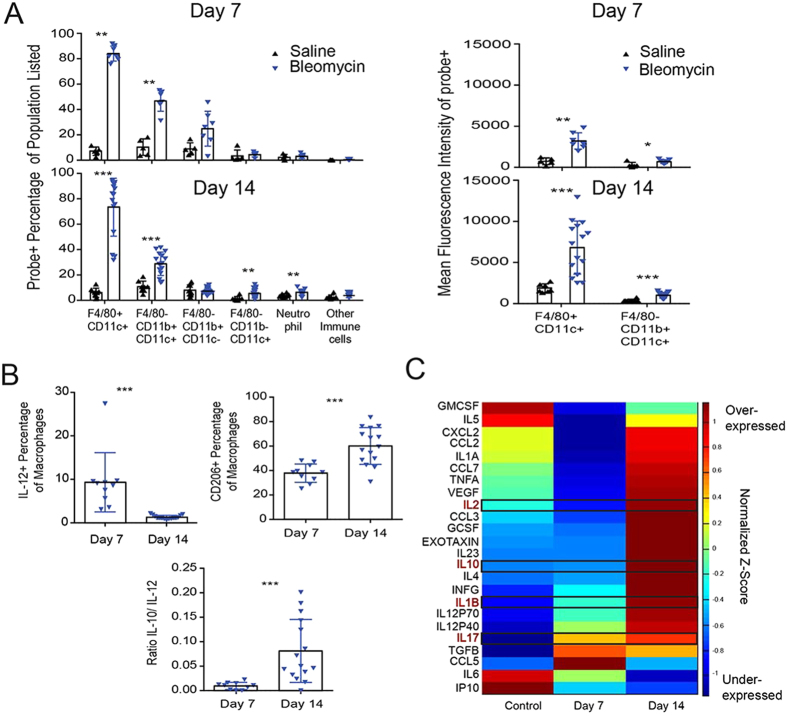
Immunologic profiling during progression of pulmonary fibrosis. (**A**) Analysis of Day 7 and 14 BAL fluid from mice to monitor activated macrophages during disease progression. Samples were analysed by FACS for the indicated immune cell markers. Error bars indicate mean ± SD., Day 7 (saline n = 5; bleomycin n = 7) and Day 14 (saline n = 8; bleomycin n = 15). *p < 0.05, **p < 0.01, ***p < 0.005 by Mann-Whitney test. (**B**) Intracellular flow cytometric analysis of macrophages expressing IL-10, IL-12 and CD206, measured in BAL fluid of bleomycin treated mice on days 7 and 14. Error bars indicate mean ± SD., Day 7 (saline n = 5; bleomycin n = 10) and Day 14 (saline n = 8; bleomycin n = 15). ***p < 0.005 by Mann-Whitney test. (**C**) Luminex cytokine profiling of plasma from Day 7 (n = 10) and Day 14 (n = 15) mice compared to control mice, pooled from Day 7 (n = 10) and Day 14 (n = 10). Colours represent level of expression of each of the indicated cytokines with red being highly expressed and blue being lowly expressed (scale bar shows z-score, standardized across each cytokine, where mean = 0, and standard deviation is 1). Several of the key cytokines linked to IPF in humans are highlighted with black boxes.

Brief exposure to bleomycin causes epithelial cell apoptosis, activating an inflammatory wound-healing response, ultimately leading to pulmonary fibrosis. We sought to further characterize the immune cell infiltrate and define the distinct cell populations labelled by BMV109 during bleomycin-induced fibrosis. After bleomycin exposure, probe labelling in the lung co-localized specifically with the macrophage marker CD68, with the intensity of the probe signal increasing from Day 7 to Day 14 as disease burden increased (Supplementary Fig. S2C). Flow cytometric analysis allowed us to further characterize the population of infiltrating leukocytes in diseased lung, with particular focus on the probe labelled macrophage population. Bronchoalveolar lavage (BAL) fluid from mice post bleomycin exposure had significantly more leukocytes compared to saline controls. Macrophage populations dominated the immune infiltrate across all groups tested. Neutrophils were enriched in control and bleomycin treated mice on day 7, but declined by day 14. Day 14 was marked by a significant increase in other immune cells, consistent with lymphocytes (by forward and side scatter profiles), corresponding to increased inflammation (Supplementary Fig. S2D). BMV109 staining was highly enriched in the F4/80+/CD11c+ macrophage populations, with the intensity of probe per cell (mean fluorescence intensity, MFI) greater in bleomycin-treated mice at both time points, indicating increased cathepsin activity in these cells (Fig. 3A). Intracellular flow cytometric analysis revealed an increased macrophage expression of pro-inflammatory IL-12 at day 7, in BAL fluid that was not sustained at day 14 (Fig. 3B). Furthermore, a shift to the pro-fibrotic response was evident, with increased M2-like macrophage associated IL-10 production and CD206 expression.

Coordinate or discordant regulation of several cytokines and chemokines are reported to correlate with IPF disease progression^30^. To determine if these markers mimicked levels observed in the human condition, we performed multiplexed cytokine profiling of plasma from bleomycin-treated mice at Day 7 and Day 14 (Fig. 3C). We found that the cytokines IL-1β, IL-10 and IL-2 increased from Day 7 to Day 14. Similarly, a downstream target of IL-1β, IL-17 increased from the early to late stages of the disease. This cytokine plays a role in mediating the fibrotic response^31^. Markers of the Th2, pro-fibrotic immune response, IL-4 and IL-5, were low at Day 7 but increased with disease progression at Day 14. Overall, the changes in the levels of cytokines and chemokines upon induction of fibrosis in the mouse model were consistent with those observed in human IPF samples^32^,^33^.

### PET/CT and optical imaging of cathepsin activity in the bleomycin lung fibrosis model

**Figure 4:**
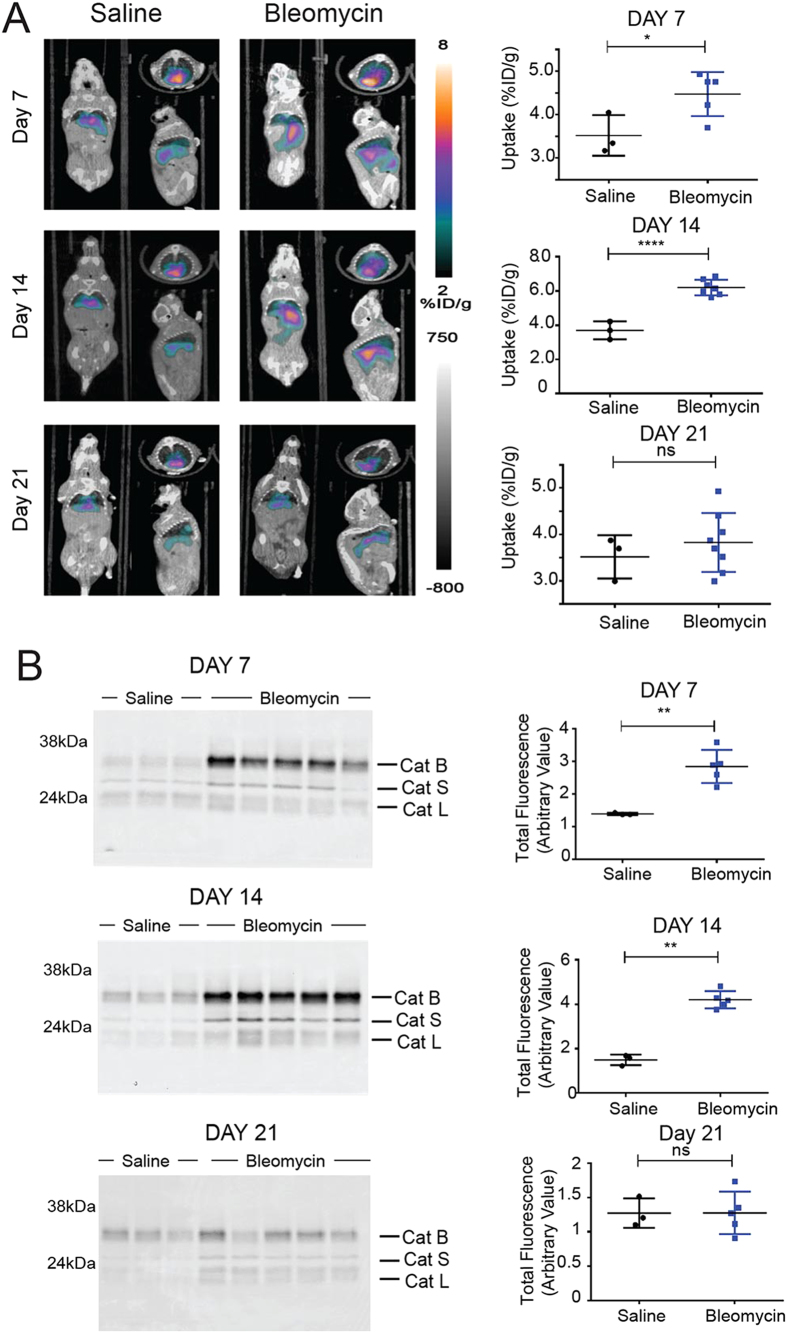
Application of the ABP ^64^Cu-BMV101 as a dual optical/PET imaging probe. (**A**) Non-invasive PET/CT scans of mice treated with saline or bleomycin at Day 7, Day 14 and Day 21. Coronal (left), transaxial (top right) and sagittal (bottom right) images are shown for representative mice from saline or bleomycin treated groups at the indicated time points. Corresponding quantification of PET/CT intensity from lungs of all mice at Day 7, Day 14 and Day 21 in the different treatment groups are shown alongside PET/CT images. Error bars indicate mean ± SEM., Day 7 (saline n = 3; bleomycin n = 5), Day 14 (saline n = 3; bleomycin n = 7) and Day 21 (saline n = 3; bleomycin n = 8). *p < 0.05, ****p < 0.0001 by *t*-test. (**B**) SDS-PAGE analysis followed by flatbed laser scanning to detect probe labeled cathepsins in lung tissue lysates from mice from the different treatment groups (saline n = 3 and bleomycin n = 5). Total cathepsin labeling intensity was measured using imageJ and plotted for each group. Error bars indicate mean ± SEM., **p < 0.005 by *t*-test. The identity of specific cysteine cathepsins is indicated for the SDS-PAGE images. For full uncut gel images see supplementary information files.

We next applied ^64^Cu-labeled BMV101 to the bleomycin model. We chose this radionuclide because it has a relatively long half-life (12.7 hrs) and therefore could be used for imaging at extended time points (i.e. 24 hr) after probe injection. We performed optical/PET-CT imaging studies with ^64^Cu-BMV101 at the same time points post bleomycin treatment that we used for the optical probe except we added a Day 21 time point to cover later stage progression of the disease (Fig. 4 and Supplementary Fig. S2,3). Again, disease burden was evident in the bleomycin treated groups as measured by total body weight loss and increased lung weight (Supplementary Fig. S3A). We performed small animal PET-CT imaging and observed increased probe signal at Day 7 and Day 14 in lungs of bleomycin treated mice relative to control mice (Fig. 4A). Furthermore, this level increased from Day 7 to Day 14 as macrophage infiltration increased. At Day 21, we observed reduced probe signal in the bleomycin treated lungs, likely due to the fact that in the rodent fibrosis model, immune cell infiltration decreases over time^34^. Biodistribution studies confirmed that the probe accumulated in the blood, liver, heart and kidney, but showed the most significant increased accumulation in the lungs of the bleomycin treated mice compared to controls (Supplementary Fig. S5A). Specific probe uptake into lungs was confirmed by quantification and comparison of lung to liver and lung to muscle ratios at each time-point (Supplementary Fig. S5B). Since the probe has dual optical and PET labels, we were also able to visualize probe signals in the lungs by non-invasive optical imaging and by *ex vivo* imaging of the lungs (Fig. S4). Day 7 and Day 14 mice with lung fibrosis showed significantly higher optical probe signal in the lung when compared to the controls by both non-invasive and *ex vivo* imaging (Supplementary Fig. S4), consistent with the PET-CT results. Subsequent analysis of lung tissues by SDS-PAGE revealed increased levels of active cathepsins, dominated by cathepsin B and S, in the bleomycin-induced lung samples relative to the controls (Fig. 4B). Non-invasive and *ex vivo* analysis of Day 21 samples showed decreased lung cathepsin expression, which correlated with a decrease in macrophage infiltration (Supplementary Fig. S4). This was further confirmed by histology and immunofluorescence analysis (Supplementary Fig. S3B and S3C). Taken together, these results confirm that the non-invasive PET imaging signals correlated with the onset of fibrosis at days 7 and 14 and correlated with the presence of activated macrophages in the lungs.

### Topical labelling of human lung tissues from patients with idiopathic pulmonary fibrosis

**Figure 5:**
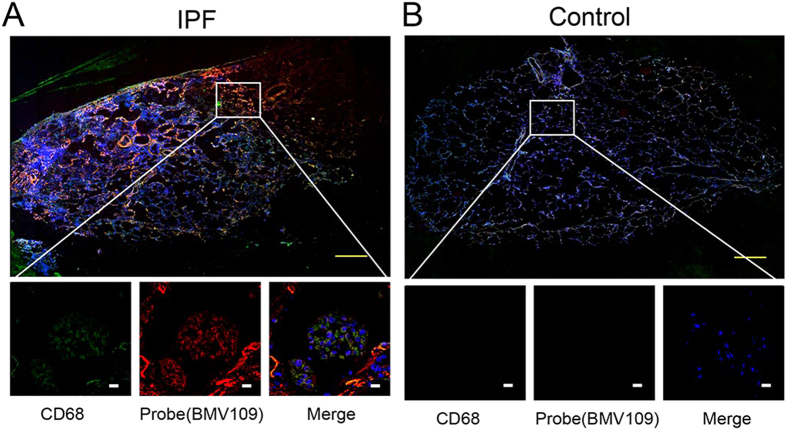
Topical staining of human tissues with BMV109. Representative images from IPF (**A**) and healthy control (**B**) human lungs that were labelled with the optical probe BMV109 (red) and co-stained with the marker for macrophages, CD68 (green). Samples were tile scanned at high resolution to generate full images. Insets show regions of interest at higher magnification. White scale bars on zoom images are 10 μm. Yellow scale bars on full tiled images represent 1 mm. Additional patient sample data in Supplementary Fig. S6.

We have recently shown that small molecule ABPs can be used to topically label active cathepsins in excised tissues^21^,^35^. We therefore topically labelled sectioned frozen tissue samples from IPF patients undergoing surgical lung biopsy or lung transplantation with the optical probe BMV109 (Fig. 5, S6). We also co-stained the tissues with the macrophage marker (CD68). These data confirmed labelling of macrophages that are actively expressing cysteine cathepsins and that the signals for active cathepsins were specifically found at sites of fibrotic lesions. Thus, cathepsins may be ideal biomarkers for monitoring of disease activity in IPF.

### Application of an activity based PET imaging probe in patients with idiopathic pulmonary fibrosis

We chose to use ^68^Ga as our radionuclide for first-in-human trials since it has been used extensively in clinical imaging and is relatively easy to generate. However, it has a short half-life (68 min) and therefore required imaging within the first few hours of probe administration. Prior to translation into studies in humans, we tested the effectiveness of ^68^Ga-BMV101 in the bleomycin lung fibrosis model (Supplementary Fig. S7). These results confirmed that it produced similar results to ^64^Cu-BMV101, with increased probe signal observed in lungs of bleomycin treated mice.

**Figure 6:**
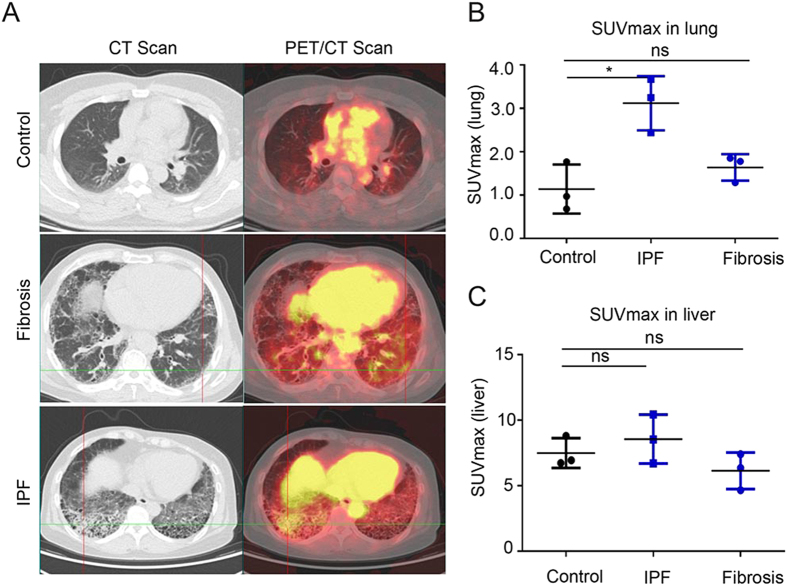
First-in-human application of imaging probe ^68^Ga-BMV101. (**A**) Representative scans of patients with idiopathic pulmonary fibrosis (IPF) and unclassifiable pulmonary fibrosis (Fibrosis) compared to healthy controls. Left hand images show axial CT image in the lung window. Right hand images show axial fused PET/CT of the same section. (**B**) Plot of quantitative SUVmax values for lung and liver from controls, IPF and fibrosis patients. Error bars indicate mean ± SEM., *p < 0.05 by Wilcoxon Mann Whitney U test.

For the human clinical studies, we recruited six subjects and three controls with healthy lung function (Supplementary Table S1). Of the six patients with lung fibrosis, three were confirmed as having idiopathic pulmonary fibrosis by multidisciplinary review according to ATS/ERS/JRS/ALAT criteria^3^ while the remaining three were unclassifiable pulmonary fibrosis. Subjects were between the age of 51 and 76, were predominantly former smokers, and had varying levels of physiological impairment. The ^68^Ga-BMV101 PET-CT probe was injected intravenously and images were collected at 2.5 hours post probe administration. These data demonstrated that the probe showed increased accumulation in regions of fibrosis in the lungs of the three patients with IPF, with no significant increase in probe accumulation in the unclassified fibrosis patients relative to the normal lung (Fig. 6). Importantly, there was also no significant increase in probe accumulation in the liver of the patients with IPF or lung fibrosis compared to the healthy controls. Overall, the ratio of probe signals in lung compared to muscle were high (4.36 for IPF patients and 1.37 for normal controls; Table S) with more than 3 times greater accumulation of the probe in lungs of patients with IPF. Taken together, these results confirm that the probe is a viable PET contrast agent that accumulates in regions of lung fibrosis in patients with IPF. The data also suggest that the bleomycin-induced fibrosis in the mouse correlates with the pathobiology of human patients with IPF, and suggest that macrophages may be important regulators of disease progression in humans.

## Discussion

Concerted efforts have recently been made to develop new technologies for early disease monitoring to facilitate diagnosis and therapeutic intervention for IPF. Optical and PET imaging has great promise and potential application in this respect^22^. Activity based probes (ABPs) generate a signal in response to covalent modification of a specific enzymatic target and are therefore useful as contrast agents for imaging. Cysteine cathepsins expressed in antigen presenting cells such as macrophages, are promising biomarkers of macrophage activation, with cathepsins B and S in particular, implicated in lung disease^36^,^37^. Recent evidence suggests that these immune cells play important roles in the overall disease pathology of IPF^8^. For these reasons, we hypothesized that ABPs that target cysteine cathepsins would specifically highlight infiltrating macrophages that contribute to the pathobiology of IPF, and that these ABPs could have broad value for non-invasive monitoring of disease activity. We further expect that these probes might also allow for early diagnosis with increased sensitivity compared to HRCT^38^. Currently, there have been no reported non-invasive imaging clinical studies of any contrast agent in patients with IPF.

Here we describe the application of cysteine cathepsin activity-based probes to the rodent model of lung fibrosis and in human IPF lungs. The relevance of the bleomycin mouse model to IPF has been questioned because it is thought to mimic acute lung injury followed by scar formation, rather than abnormal wound healing or progressive fibroproliferation. Also, with the direct administration of bleomycin to the lungs, the initial phase of bleomycin-induced injury is primarily pro-inflammatory with an influx of neutrophils and mononuclear infiltrate^30^, which has not been observed in IPF. Although the bleomycin model has been criticized for its speed of disease onset and durability of fibrosis, it remains a robust, well-established, reproducible model of lung fibrosis that can reveal potential mechanisms underlying the human disease^30^,^31^. Our results presented here further confirm that this model produces many of the same hallmarks of human IPF disease pathology, especially with respect to the activation and accumulation of macrophages and the production of hallmark cytokines. Specifically, our data shows that IL-1β, IL-10, IL-2, were key cytokines that increased from Day 7 to Day 14 in the murine model, consistent with what has been observed in primary patient samples^9^. Thus, for the purposes of validating imaging probes that aim to report on relevant aspects of the immune program in IPF disease pathogenesis, this model remains highly valuable.

The first-in-human results for the ^68^Ga-labeled probe provide strong support for the notion that activated macrophages are regulators of the pathobiology of IPF disease progression. Our clinical results mirror our findings in the bleomycin mouse model of fibrosis where we see accumulation of macrophages with high cathepsin activity both at early onset and once the fibrotic process has been fully established. This again suggests that the bleomycin model proves valuable information that is relevant to the human disease, with respect to immune cell involvement. Furthermore, the fact that we do not see significant probe accumulation in patients with unclassified lung fibrosis provides some support for the potential to use cathepsin probes to stratify patients with IPF such that they can be followed and eventually treated with new therapies that are currently in clinical trials. We believe that additional follow up imaging trials on a larger number of patients, coupled with collection of BAL fluids for analysis of levels of activated macrophages in the lungs, will allow a more clear understanding of the importance of activated macrophage populations with respect to IPF disease severity, progression and response to treatment.

While our initial results with the dual optical/PET probe are promising both in the preclinical animal models and in human patients with IPF, we noted that the overall signal to noise ratios were rather poor (see Tables S2-4). Specifically, when comparing lung to liver signals we obtained values below a value of 1. This is likely do to high baseline cathepsin activity in the liver and also relatively high blood pooling of the probe. This explanation was further supported by the fact that lung to muscle ratios were substantially higher. We also observed high PET signals in the blood (Fig. S5). This blood pooling effect is likely due to the highly hydrophobic/aromatic nature of the probe. Therefore, we are currently working to identify a more effective probe backbone structure that is still recognized by the protease targets but that is more highly hydrophilic and charged. This should both help to reduce the blood retention of the probe and also increase solubility and formulation properties which should facilitate further clinical studies.

In conclusion, we report the development of a new bimodal optical/PET probe and its use as an imaging agent for monitoring the pathobiology of IPF. Having a dual label on the probe allows for initial PET imaging and subsequent optical analysis by *in vivo* and *in vitro* methods. Importantly, the use of a PET/optical probe, for the first time, provided a means to non-invasively image regions of lung fibrosis and correlate findings in the mouse model of fibrosis with human IPF disease. We find that the early stages of fibrosis in the mouse model involve macrophage responses that peak early in the onset of fibrosis (i.e. day 15). In humans, we find that the probe accumulates in regions of fibrosis that are more likely late events that may involve persistent, unresolved injury around established fibrotic lesions. Thus, our probe could be highly valuable for assessing overall disease activity, even in patients who have advanced disease. Furthermore, these results suggest that our probe has the potential to be applied in the clinic to facilitate study of the cellular and molecular events that drive IPF pathology. They are also likely to be useful preclinical and clinical tools for other diseases that involve contributions from activated immune cells that express the cysteine cathepsins.

## Materials and Methods

### Synthesis of Probes

Details of the probe synthesis, radiolabeling and compound characterization can be found in the supplemental methods section.

### Animals and Model Induction

Female C57BL/6J mice weighing 20–25 g were purchased from Jackson Laboratories (Bar Harbor, ME). To induce pulmonary fibrosis, mice were treated on day 0 with 1.5 unit/kg body weight of bleomycin (MP Biomedicals LLC) by endotracheal instillation as previously described^39^; control animals received sterile saline. At the indicated times (day 7 and day 21), the probe BMV109 was injected at a dose of 10 nmol in 10% DMSO/PBS, 4 hours prior to harvest, and mice were imaged non-invasively using the IVIS 100 system. Animals were sacrificed under isoflurane anesthesia and BAL fluid extracted as previously describe^40^. Lungs were then removed, imaged *ex vivo* using an FMT system. Samples of lung tissue and cells were collected for further analysis, as described below. A total of 10 animals were used for each time point, divided equally between control and bleomycin-treated groups and not randomized. This sample size was chosen based on knowledge that some animals may not survive the course of the experiment and that a cohort of at least 3 animals per group are required in order to get statistical significance in the results. Investigators conducting the trial were not blinded as to what groups were being studied unless otherwise stated. All mouse experiments were approved by the Stanford Administration Panel on Laboratory Animal Care and strictly followed their specific guidelines.

### Murine PET *In Vivo* Imaging

Mice were intravenous injected with 100 μCi of ^64^Cu-BMV101 and imaged after 1 h, 2 h, and 4 h by using Inveon small-animal PET/CT (Siemens). Briefly, a CT anatomic image scan was acquired (80 kV, 500 μA) with a pixel size of approximately 0.1 mm. After CT imaging, whole-body PET imaging was performed with 5 min static scan. The PET images were reconstructed using the ordered-subsets expectation maximization 3-dimensional algorithm based on CT attenuation and analyzed using the Inveon Research Workplace (IRW) software (Siemens). PET voxel size was 0.796 × 0.861 × 0.861 mm, for a total of 128 × 128 × 159 voxels. After the 4 h PET/CT imaging, the mouse was injected with 100 μL BMV101 (10 mM) and imaged for fluorescence signal by IVIS200 (Perkin Elmer) at 1 h later. Then PET/CT quantify assay was performed, and tissue radioactivity was calculated and expressed as decay-corrected percentage injected dose per gram of tissue (%ID/g). Investigators conducting the trial were blinded as to what groups were being imaged.

### Tissue and cell labeling and analysis

Details of the tissue and cell preparation, probe labeling and analysis by microscopy and flow cytometry can be found in the supplementary methods section.

### Patient Recruitment

This study was approved by the Institute Review Boards of Peking Union Medical College Hospital and conducted from December 2014 to April 2015 and was carried out in accordance with the approved guidelines. Nine patients were enrolled in this study after obtaining written informed consent. The subjects were deemed to have IPF or IPF-related condition based on their clinical history, physical examination, standard blood and urine tests, CT and electrocardiogram. Subjects were contacted by telephone approximately 24 h after ^68^Ga-BMV101 administration, for adverse events monitoring. Subjects included were between the ages of 35 and 80 and had fibrotic interstitial lung disease as defined by the presence of reticulation, traction bronchiectasis and honeycombing on high-resolution computed tomography (HRCT) imaging of the chest. Controls without lung disease were recruited from patients undergoing PET-CT scans for non-pulmonary indications. Exclusion criteria included pregnancy, lactation, the presence of co-existing emphysema on HRCT, and impaired renal or liver function. Human subjects research was approved by the ethics review committees of Stanford University Medical Center (Stanford, CA, United States) and Peking Union Medical College (Beijing, China), and all participants provided informed consent.

### Statistical Analysis of Data

Statistics were performed using the data analysis package within GraphPad Prism 6.0 for Windows (GraphPad Software, San Diego, CA, USA). For the human studies, SUVmax values from subjects and controls were compared using the Wilcoxon Mann Whitney U test to account for non-normally distributed data. Statistical significance was defined as a p value < 0.05. Unless otherwise stated, tests comparing two means are a Student’s *t*-test, with equal variance assumed. Error bars indicate standard error of the mean (SEM) unless otherwise stated.

## Additional Information

**How to cite this article**: Withana, N. P. *et al*. Non-invasive Imaging of Idiopathic Pulmonary Fibrosis Using Cathepsin Protease Probes. *Sci. Rep*. **6**, 19755; doi: 10.1038/srep19755 (2016).

## Supplementary Material

Supplementary Information
