# Supplementary material for: Non-invasive Imaging of Idiopathic Pulmonary Fibrosis Using Cathepsin Protease Probes
Source: Sci Rep. 2016 Jan 22;6:19755. doi: 10.1038/srep19755 (PMC4726431; doi:10.1038/srep19755)
Supplement: Supplementary Information [file srep19755-s1.doc]

Supplementary Data and Figures

**Non-invasive Imaging of Idiopathic Pulmonary Fibrosis Using Cathepsin Protease Probes**

Nimali P. Withana^1#^, Xiaowei Ma^2#^, Helen M. McGuire^3^, Martijn Verdoes^1,*^, Wouter A. van der Linden^1^, Leslie O. Ofori^1^, Ruiping Zhang^2^, Hao Li^4^, Laura E. Sanman^4^, Ke Wei^5^, Shaobo Yao^6^, Peilin Wu^6^, Fang Li^6^, Hui Huang^7^, Zuojun Xu^7^, Paul J. Wolters^8^, Glenn D. Rosen^5^, Harold R.Collard^8^, Zhaohui Zhu^6**^, Zhen Cheng^2**^and Matthew Bogyo^1,2,3**^

Supplemental Methods:

**Synthesis of the BMV101 probe**

Compound **2**. Boc-Lys-OH (1 g, 4 mmol) was dissolved in chloroform/DMF (2/1), trityl bromide (2.84 g, 8.8 mmol, 2.2 equiv.) was added and stirred till the solution became clear. Triethylamine (2.24 ml, 16 mmol, 4 equiv.) was added dropwise over 20 min and stirred for an additional 30 min. Methanol was added (5 ml) and the reaction mixture was stirred at 50 °C for 2 hr, before being concentrated *in vacuo*. The crude was taken up in diethyl ether, washed with 10% citric acid and water, dried over Na_2_SO_4_ and purified by flash chromatography (Hexane → 50% Ethyl acetate in hexane).

Compound **3**. To a solution of compound **2** (51.2 mg, 105 μmol, 1.4 equiv.), HBTU (39.8 mg, 105 μmol, 1.4 equiv.), DiPEA (45.5 μl, 261 μmol, 3.6 equiv.) in DMF was added GB111-NH_2_ ∙ TFA (50 mg, 73 μmol) and the reaction mixture was stirred overnight, before being concentrated *in vacuo*. The crude material was purified by flash chromatography (Hexane → 40% Ethyl acetate in hexane). Yield: 80 mg (73 μmol, quant.).

Compound **4**. Compound **3** (10 mg, 9.6 μmol) was taken up in 1% TFA in DCM and stirred for 15 min, before a drop of water was added and the reaction mixture was coevaporated with toluene (3x). The crude was taken up in DMF and NOTA (60 μmol, 6.25 equiv.), HBTU (68 mg, 180 μmol, 18.75 equiv.) and DiPEA (34.8 μl, 200 μmol, 20.83 equiv.) were added and the reaction mixture was stirred overnight, before being concentrated *in vacuo*. The crude was used without further purification.

**BMV101** (**5**). Crude **4** (~0.96 μmol) was taken up in DCM/TFA (1/1) and stirred for 30 min, before being coevaporated with toluene (3x). The crude was taken up in DMF and DiPEA (0.9 μl, 5 μmol, 5 equiv.) and Cy5-NHS (0.75 mg, 1 μmol, 1 equiv.) were added. After 2 hr the reaction mixture was purified by HPLC (small column, 20-55% (20CV)). Yield: 0.91 mg (0.56 μmol, 58%).

**Radiolabeling of BMV101**

^64^Cu-BMV101 was prepared by incubation of 10μL BMV101 (10mM) in 90μL sodium acetate buffer (0.1M, pH 5.5) with 4 mCi of ^64^CuCl_2_ in 100μL sodium acetate buffer (0.1M, pH 5.5) at 37 ℃ for 1 hour. After cooling to room temperature, the reaction mixture was analyzed by RP-HPLC. The ^64^Cu-BMV101 was then purified by RP- HPLC with the mobile phase starting from 95% solvent A (di-water with 0.1% TFA) and 5% solvent B (acetonitrile with 0.1% TFA) for 3 min to 5% solvent A and 95% solvent B at 23 min. The eluted fractions containing ^64^Cu-BMV101 (retention time 18.9 min) were then collected and dried using a rotary evaporator. The radiolabeling yield was 90% (calculated from the HPLC). The radiochemical purity, defined as the ratio of the main product peak to other peaks, was determined by radio-HPLC to be >95%, and the specific activity of the probe was determined to be 3-4 Ci/mmol. The ^64^Cu-BMV101 was then reconstituted in 0.9% saline and passed through a 0.22 μm Millipore filter into a sterile vial for animal PET/CT imaging.

^68^Ga-BMV101 was synthesized immediately before the injection in a hot cell using a manual method. Briefly, 1 mL ^68^GaCl_3_ eluent (370-555 MBq, pH 2.0-3.0) obtained from a ^68^Ge/^68^Ga generator in a 1.5 mL centrifuge tube was added 93 μL sodium acetate solution (NaOAc, 1.25 M) to adjust the pH range from 3.5 to 4.0. After addition of 20 μL of BMV101 (1 μg/μL in DMSO) to the mixed solution was heated to 37 °C for 30 min. After cooling to room temperature, the reaction mixture was diluted to 5 mL and passed through a preconditioned Sep-Pak C18 Plus Light Cartridge at a rate of one drop per second. The C18 cartridge was further washed by water (0.5 mL × 2) to remove the excess ^68^Ga ion and other water soluble materials. The resulting solution was analyzed by HPLC chromatography (Waters, USA). The mobile phase starting from 5% solvent A (acetonitrile with 0.1% TFA) and 95% solvent B (di-water with 0.1% TFA) for 5 min; ramped to 80% solvent A and 20% solvent B to 10 min; ramped to 100% solvent A and 0% solvent B to 15 min; the ratio was kept to 18 min; then ramped to 5% solvent A and 95% solvent B to 20 min. The ^68^Ga-BMV101 was then reconstituted in 0.9 % saline and filtered through a 0.2 μm Millex-LG filter (EMD Millipore) into a sterile vial before use.

**Radiopharmaceutical Preparation**

^68^Ga (half-life is 68 minutes, energy of β+ is 1.9 MeV) was eluted from the ^68^Ge/^68^Ga generator (ITG, Germany) with 0.1 M HCl. The major radioactivity peak was collected as 2 mL final volume and 20 mCi. Then, the pH was adjusted to 4.5 with 1.25 M sodium acetate buffer (pH 4.5). 10 μL BMV101 (1mM in DMSO) was added to the ^68^GaCl_2_ elution and the mixture was incubated at 42 ºC for 30 minutes. The radiochemical yield was analyzed via RP-HPLC and thin layer chromatography (iTLC, Bioscan, USA). The product was injected through C_18_ light cartridge and was eluted with 200 μL ethanol. The elution was diluted with 3 mL of 0.9% NaCl and passed through a 0.22 μm filter. For clinical study, this whole radiolabeling process was done in a good manufacturing practice (GMP) environment. The stability of ^68^Ga-BMV101 in mouse serum was evaluated by incubating 500 μCi ^68^Ga-BMV101 and 500 μL mouse serum for 3 hours. For the biodistribution assays in mice, a series of PET scans were performed at 1 h and 2 h respectively after intravenous injection of 3.7−7.4 MBq ^68^Ga-BMV101. For clinical evaluation, 3 normal volunteer and 6 fibrotic interstitial lung disease (ILD) patients were injected with ^68^Ga-BMV101 as 1.2 MBq per kilogram body weight. Whole-body PET images were acquired at 2.5 hours after intravenous injection of ^68^Ga-BMV101. The scan parameters were 140 kv, 35 mA, pitch 1: 1, thickness of 3 mm, spaced 3 mm, matrix512 × 512, and FOV 70 cm. PET / CT examination was done using the Siemens Biograph 64 Truepoint TrueV PET / CT. The standardized uptake value (SUV) of lung, liver, and muscle were measured for semi-quantitative evaluation.

**Flow Cytometry.**

Cells obtained from the extracted BAL fluid were subjected to flow cytometric analysis. Antibodies purchased from Biolegend were (F4/80 Cat#123119, Ly-6G Cat#127611, CD68 Cat#137013, CD11c Cat#117323, and CD11b Cat #101237). Cells were acquired using a LSRII cytometer (BD Biosciences, San Diego, CA) and analyzed using FlowJo (Tree Star, Ashland, OR). Intracellular cytokines were detected after 4 hours stimulation at 37^o^C (1 μg/ml LPS, and GolgiStop; 1:1000, BD Biosciences) using BD Biosciences intracellular staining kit according to the manufacturer’s instructions.

**Plasma Cytokine Detection with Luminex (Polystyrene bead kit).**

Blood collected into tubes containing 200 ul EDTA/heparin were subjected to centrifugation at 2.8 K RPM RCF for 5 min and the supernatant removed, aliquoted into cryovials, and frozen at -80^o^C. Plasma samples were assayed with the Mouse 26 plex kit purchased from Affymetrix, and performed in the Human Immune Monitoring Center at Stanford University according to the manufacturer’s recommendations with modifications as described previously{Rosenberg-Hasson, 2014 #153}. Results were shown by heatmap with values normalized by z-scores.

**Lysate Labeling.**

RAW cells were seeded in six-well plates (typically 1.5× 10^6^/well) 24 h prior to harvest. Cells were then washed once with PBS, scraped, and pelleted. Cells were then lysed in acidic buﬀer (50 mM sodium citrate [pH 4.5], 0.1% CHAPS, 1% NP-40, and 4 mM DTT), snap-frozen in liquid N2, and centrifuged at 4 °C for ten minutes. Supernatants were collected, and protein concentration was determined using a BCA kit. An amount of 50 μg of total protein was then aliquoted, followed by addition of the indicated probe from a 100× DMSO stock solution, yielding a ﬁnal DMSO concentration of 1%. Samples were incubated for 30 min at 37 °C and then solubilized with 4× sample buffer. Proteins were resolved by 12% SDS-PAGE and scanned using a Typhoon ﬂatbed laser scanner (excitation 633 nm/ emission 670 nm). For the pH dependence assay, citrate buffer was adjusted to the indicated pH just prior to lysis. Cells were tested for mycoplasma contamination prior to conducting experiments.

**Intact Cell Labeling.**

RAW cells were seeded as above 24 h prior to labeling. Media were replaced, and a probe was added from a 1000× DMSO stock solution, yielding a ﬁnal DMSO concentration of 0.1%. Cells were incubated for 1 h at 37 °C unless otherwise noted, washed once with PBS, scraped, and pelleted. Cells were then lysed in hypotonic lysis buﬀer (50 mM PIPES [pH 7.4], 10 mM KCl, 5 mM MgCl_2_, 2 mM EDTA, 4 mM DTT, and 1% NP-40), snap-frozen, and centrifuged at 4°C. Supernatants were then solubilized with 4× sample buffer, and 50 μg of total protein was analyzed by SDS-PAGE as above. Cells were tested for mycoplasma contamination prior to conducting experiments.

**Live Cell Imaging.**

RAW cells were seeded at a density of 100,000 cells in 35mm glass bottom dish (In Vitro Scientific). Twenty-four hours later, cells were treated with 1μM of each probe for five hours. For the last 30mins, 100 nM of lysotracker-green (Molecular Probes, L7526) was added to the cells. Cells were then washed three times with PBS, and phenol red-free complete medium was added. Cells were imaged live at 40x using a Zeiss LSM 700 confocal microscope in both Cy5 and FITC channels. Cells were tested for mycoplasma contamination prior to conducting experiments.

**Tissue Labeling.**

Tissues were sonicated in muscle lysis buﬀer (1% Triton X-100, 0.1% SDS, 0.5% sodium deoxycholate, 4 mM DTT, PBS [pH 7.4]) and analyzed as above.

**Collagen Assay.**

Sircol collagen assay were performed according to manufacture instructions{Avasarala, 2013 #154}.

**Topical application of activity based probe**

Freshly isolated human lung tissues were frozen in OCT prior to sectioning. 5 μm thick sections were fixed for 10 min in acetone at -20°C, and sections were blocked in 1% blocking reagent (Perkin Elmer Cat#FP1020) for 1h and then stained for 1 hr with 1 mM BMV109 in PBS. Sections were washed in PBS (3x5mins) and then stained with macrophage marker CD68 (1:1000 biorad MCA1815T) overnight at 4°C. Slides were washed, and the primary antibodies were detected with Alexa Fluor 488–conjugated anti-rat IgG (Molecular probes) at room temperature for 1 hours. Finally, sections were stained with DAPI and fluorescence images acquired by confocal microscopy. Sections were imaged by tile scan and at 40× using a Zeiss Axiovert 200 M confocal microscope in Cy5, FITC and Texas-Red channels. All images were taken using a multitrack channel acquisition to prevent emission crosstalk between fluorescent dyes. Single XY, XZ plane images were acquired in 1,024 x 1,024 resolution. Images were processed as separate channels using Huygens deconvolution software or ImageJ and merged as a single image. Mosaic images of fluorescence labeling were taken using 20x objective and stitched using 15% overlay.

**Microscopy.**

OCT embedded lung tissue was sectioned at 5μm and stained for macrophage marker CD68 (1:1000 AbD serotec Cat#MCA1957). Sections were imaged at 40× using a Zeiss Axiovert 200 M confocal microscope in both Cy5 and FITC channels. Human fibrotic lung tissue samples were fresh frozen and embedded in OCT. Frozen tissue sections 5μm were laid over glass slides and stained for macrophage marker CD68 (1:1000 CD68 biorad MCA1815T followed by secondary antibody with Alexa 488, Invitrogen A21151). A solution containing BMV109 probe 1μM was then applied on the tissue and incubated 1 hour at room temperature in PBS. The tissue was then washed to remove nonbound material, and detectable label was visualized with a fluorescence microscope 40× using a Zeiss Axiovert 200 M. The tissue sampling protocol was approved by the institutional review board and all patients signed informed consent to allow tissue analysis.

**Biodistribution**

After PET/CT imaging, the mice were anesthetized and sacrificed. All of organs were collected and measured with a gamma counter. The uptake were calculated and calibrated with decay-correction, and expressed as percent of injected dose per gram of tissue (% ID/g). After radioactivity counting, the lungs, livers, hearts, kidneys and spleens were also imaged with IVIS 200 for fluorescence imaging.

**
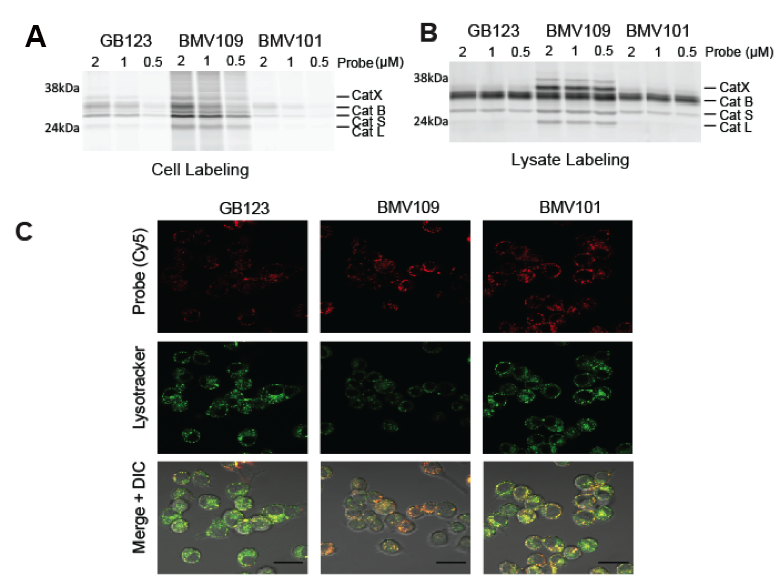
**

**Fig. S1** Labeling of raw cells with the fluorescent and PET imaging probes GB123, BMV109 and BMV101. SDS-PAGE analysis followed by flatbed laser scanning of total lysates generated from (**A**) probe labeled intact RAW cells and (**B**) lysates labeled with the indicated concentrations of GB123, BMV109 and BMV101 (**C**) Live cell images of RAW cells labeled with the indicated probes (red) and lysotracker (green) to highlight the lysosomes. Scale bars indicate 20μm. The images are representative of three independent experiments. For full uncut gel images for panels A and B see the supplemental information files.

**
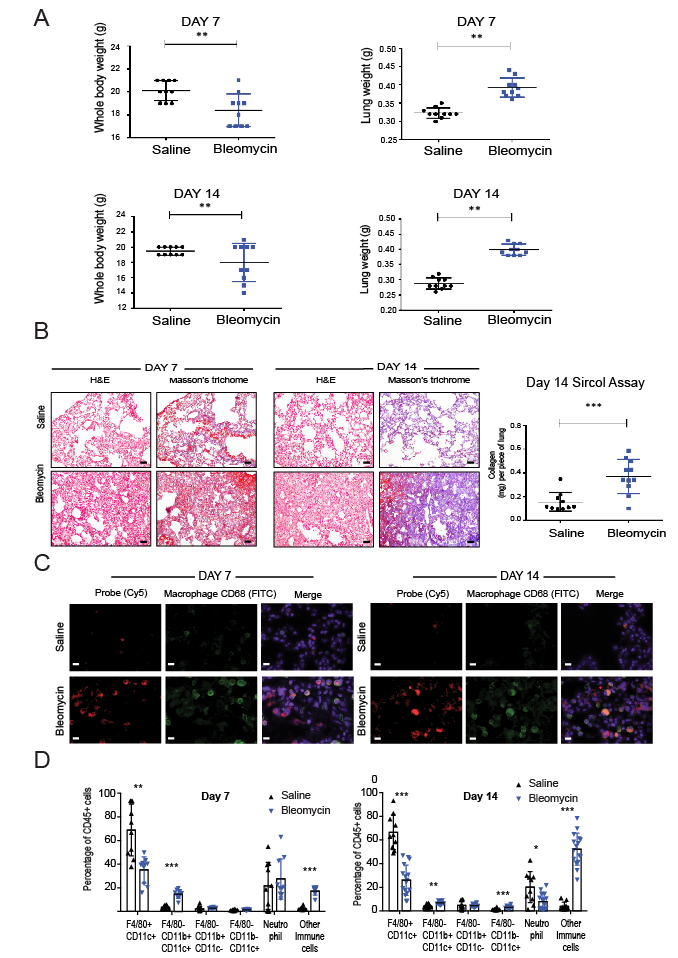
**

**Fig S2**. Confirmation of the biological outcomes from bleomycin treatment at days 7 and 14. (A) Whole body and lung weights for mice from the different treatment groups (saline vs bleomycin). (B) Histological analysis of lungs. H&E and Masson’s Trichrome Stain and Sircol Assay measuring the collagen content in the lungs of mice treated with saline or bleomycin. Scale bars indicate 10μm. **p< 0.005, ***p< 0.0001 by 2-tailed Student’s *t*-test. (C) Immunofluorescence analysis of lungs from mice treated with BMV109 (red) and co-stained with the macrophage marker CD68 (green). Scale bars indicate 10μm. (D) BAL fluid from mice to monitor activated macrophages during disease progression. Samples were analyzed by FACS. **p< 0.05, ***p< 0.005 by2-tailed Student’s *t*-test.

**
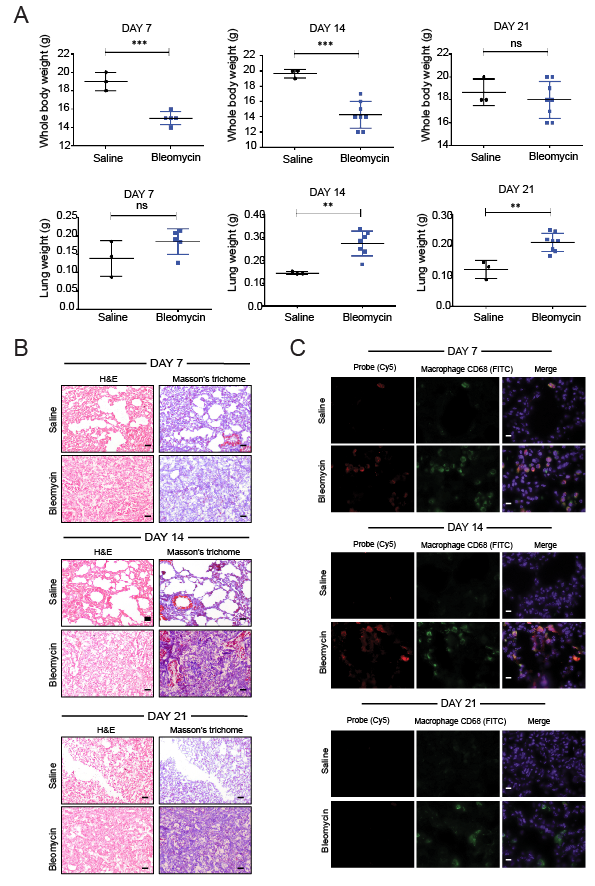
**

**Fig. S3**. Confirmation of biological outcomes of bleomycin treatment at 7, 14 and 21 days. (A) Whole body and lung weights of mice for the treatment groups (saline vs bleomycin), **p< 0.005, ***p< 0.0001 by2-tailed Student’s *t*-test. (B) Histological analysis of lungs. H&E and Masson’s Trichrome Stain. Scale bars indicate 10μm. (C) Immunofluorescence analysis of lungs from mice treated with BMV109 (red) and co-stained with the macrophage marker CD68 (green) and DAPI (blue). Scale bars indicate 10μm.


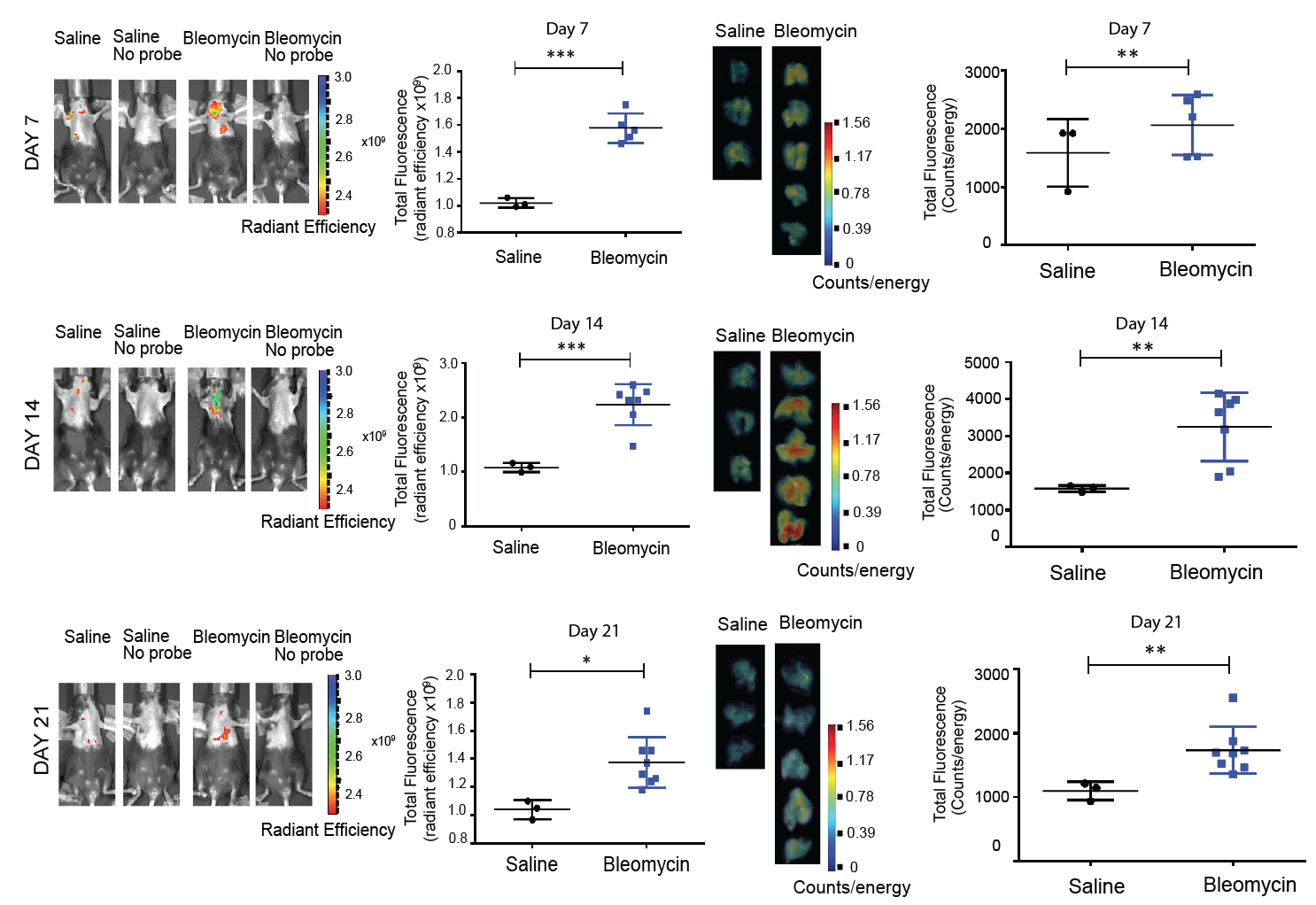


**Fig. S4**. Correlation of optical and PET/CT data. Non-invasive optical images of the same mice from figure 4 treated with saline or bleomycin (left) and corresponding ex vivo fluorescence intensity from lung (right) for the different treatment groups. Error bars indicate mean + SEM., Day 7 (saline n=3; bleomycin n=5), Day 14 (saline n=3; bleomycin n=7) and Day 21 (saline n=3; bleomycin n=8). *p < 0.05, **p< 0.005, ***p< 0.0001 by 2-tailed Student’s *t*-test.

**
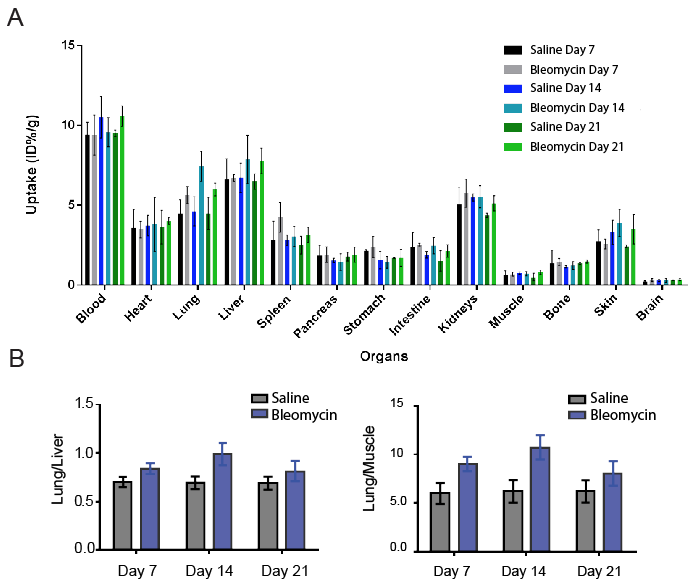
**

**Fig. S5**. PET/CT analysis on ABP ^64^Cu-BMV101 in murine model of IPF. (A) Bio-distribution of the BMV101 probe for the treatment groups at Day 7, Day 14 and Day 21. (B) Plots of average ratios (n = 3) of lung to liver and lung to muscle signals at the indicated time points.


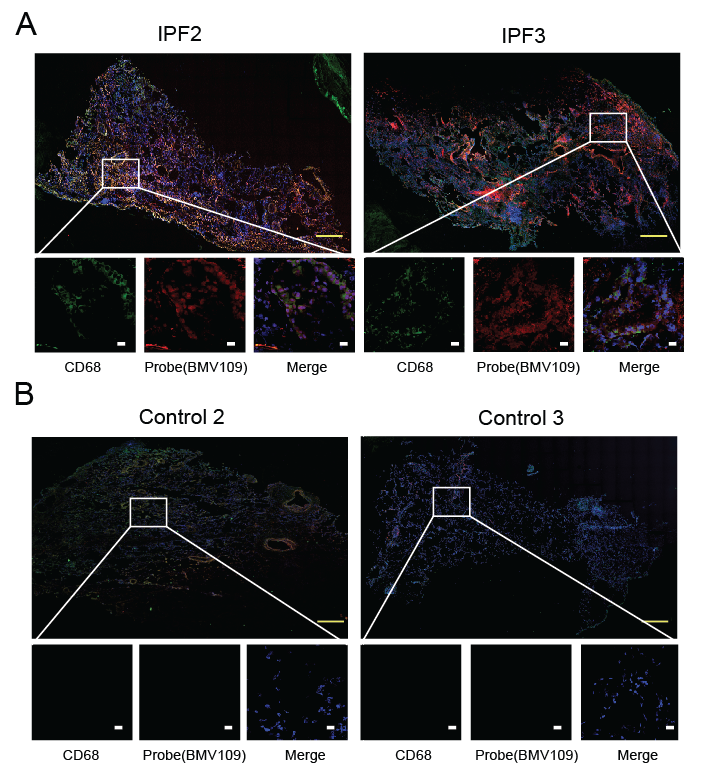


**Fig. S6.** Topical staining of human tissues with BMV109. Images from multiple IPF (A) and healthy control (B) human lung samples that were labeled with the optical probe BMV109 (red) and co-stained with the marker for activated macrophages, CD68 (green). Samples were tile scanned at high resolution to generate full images. Insets show regions of interest at higher magnification. White scale bars on zoom images are 10μm. Yellow and black scale bars on full tiled images represent 1 mm. Additional patient sample data in Fig. 5.

**
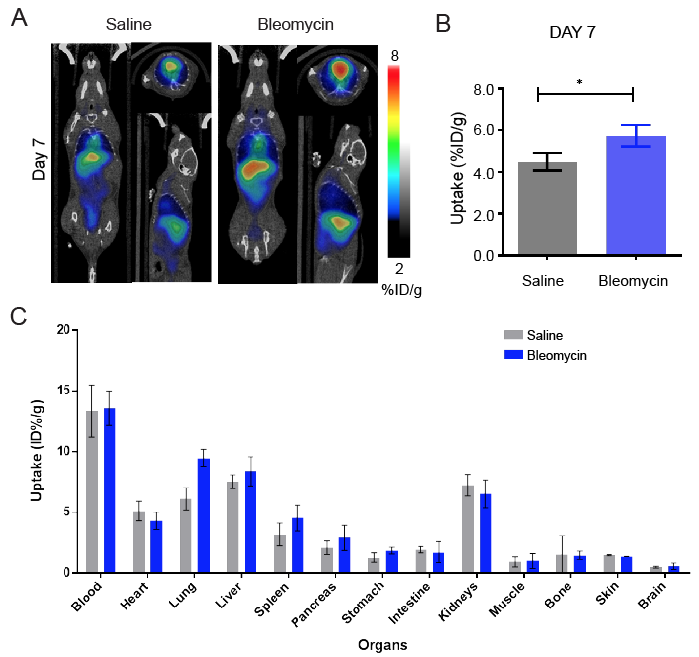
**

**Fig. S7**. Application of the ABP ^68^Ga-BMV101 as a PET imaging probe. (A) Non-invasive ^68^Ga-BMV101 PET/CT scans of mice treated with saline or bleomycin at Day 7, two hours post probe administration. Coronal (left), transaxial (top right) and sagittal (bottom right) images are shown for representative mice from saline or bleomycin treated groups. (B) Corresponding quantification of PET/CT intensity from lungs of all mice at Day 7 in the different treatment groups. Error bars indicate mean + SEM., Day 7 (saline n=3; bleomycin n=3). *p< 0.05 by2-tailed Student’s t-test. c) Bio-distribution of the ^68^Ga-BMV101 probe for the treatment groups at Day 7.

**Table S1 Values for pulmonary function test in patients with IPF, ILD and healthy controls.**

| **Subject number** | **Age** | **Sex** | **Smoking history** | **FVC**  **actual** | **FVC**  **predicted** | **FVC**  **% ratio** | **DLCO**  **actual** | **DLCO**  **predicted** | **DLCO**  **% ratio** |
| --- | --- | --- | --- | --- | --- | --- | --- | --- | --- |
| **Control-1** | 32 | F |  |  |  |  |  |  |  |
| **Control-2** | 33 | M |  |  |  |  |  |  |  |
| **Control-3** | 47 | F |  |  |  |  |  |  |  |
| **IPF-1** | 64 | M | smoked | 3.02 | 3.87 | 78.04 | 4.03 | 8.83 | 45.64 |
| **IPF-2** | 61 | M | smoked | 2.52 | 3.89 | 64.78 | 6.32 | 8.9 | 71.01 |
| **IPF-3** | 51 | M | smoked | 4.01 | 4.29 | 93.47 | 6.3 | 9.85 | 63.96 |
| **ILD-1** | 76 | F | no smoke | - | - | - | - | - | - |
| **ILD-2** | 67 | F | smoked | 2.02 | 2.46 | 82.11 | - | - | 52.30 |
| **ILD-3** | 60 | F | no smoke | 1.93 | 2.69 | 71.75 | 6.21 | 7.12 | 87.22 |

Abbreviations: FVC, forced vital capacity. DLCO, carbon monoxide (CO) diffusing capacity.

Table S2 The signal-to-noise ratios for PET/CT imaging in mice from figure 4.

|  | Lung-to-Liver | | Lung-to-Muscle | |
| --- | --- | --- | --- | --- |
|  | Saline | Bleomycine | Saline | Bleomycine |
| DAY7 | 0.57±0.02 | 0.75±0.08 | 5.74±1.14 | 8.36±0.66 |
| DAY14 | 0.62±0.08 | 1.00±0.07 | 6.34±1.20 | 10.01±1.31 |
| DAY21 | 0.58±0.11 | 0.64±0.07 | 5.73±1.23 | 7.24±1.24 |

**Table S3** The signal-to-noise ratios from the biodistribution studies from figure S5.

|  | Lung/Liver | | Lung/Musule | |
| --- | --- | --- | --- | --- |
|  | Saline | Bleomycine | Saline | Bleomycine |
| DAY7 | 0.70±0.05 | 0.84±0.06 | 5.99±1.10 | 9.00±0.72 |
| DAY14 | 0.69±0.06 | 0.99±0.11 | 6.20±1.18 | 10.71±1.27 |
| DAY21 | 0.69±0.07 | 0.81±0.11 | 6.19±1.16 | 8.04±1.23 |

Table S4 The signal-to-noise ratio for human PET/CT imaging from figure 6

|  | Lung-to-Muscle | Lung-to-Liver |
| --- | --- | --- |
| Normal | 1.37±0.62 | 0.15±0.05 |
| IPF | 4.36±1.04 | 0.37±0.07 |

Supplemental Compound Characterization:

LC/MS analysis of BMV-101


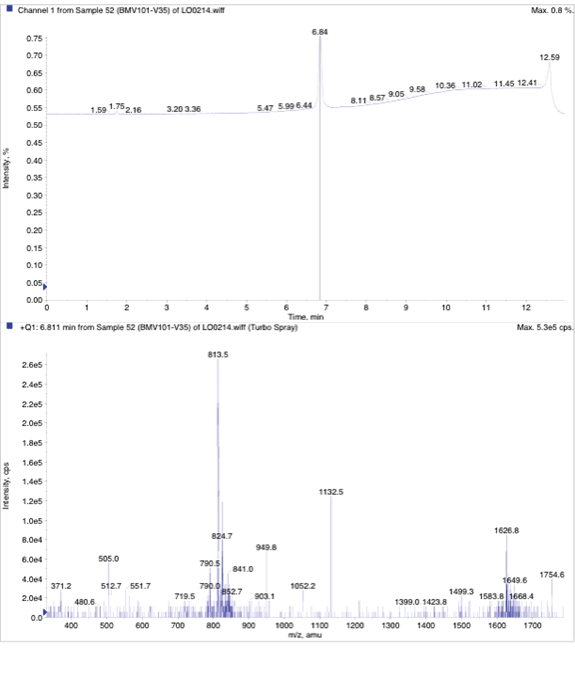


LC-MS spectra of BMV-101 without ^64^Cu label:


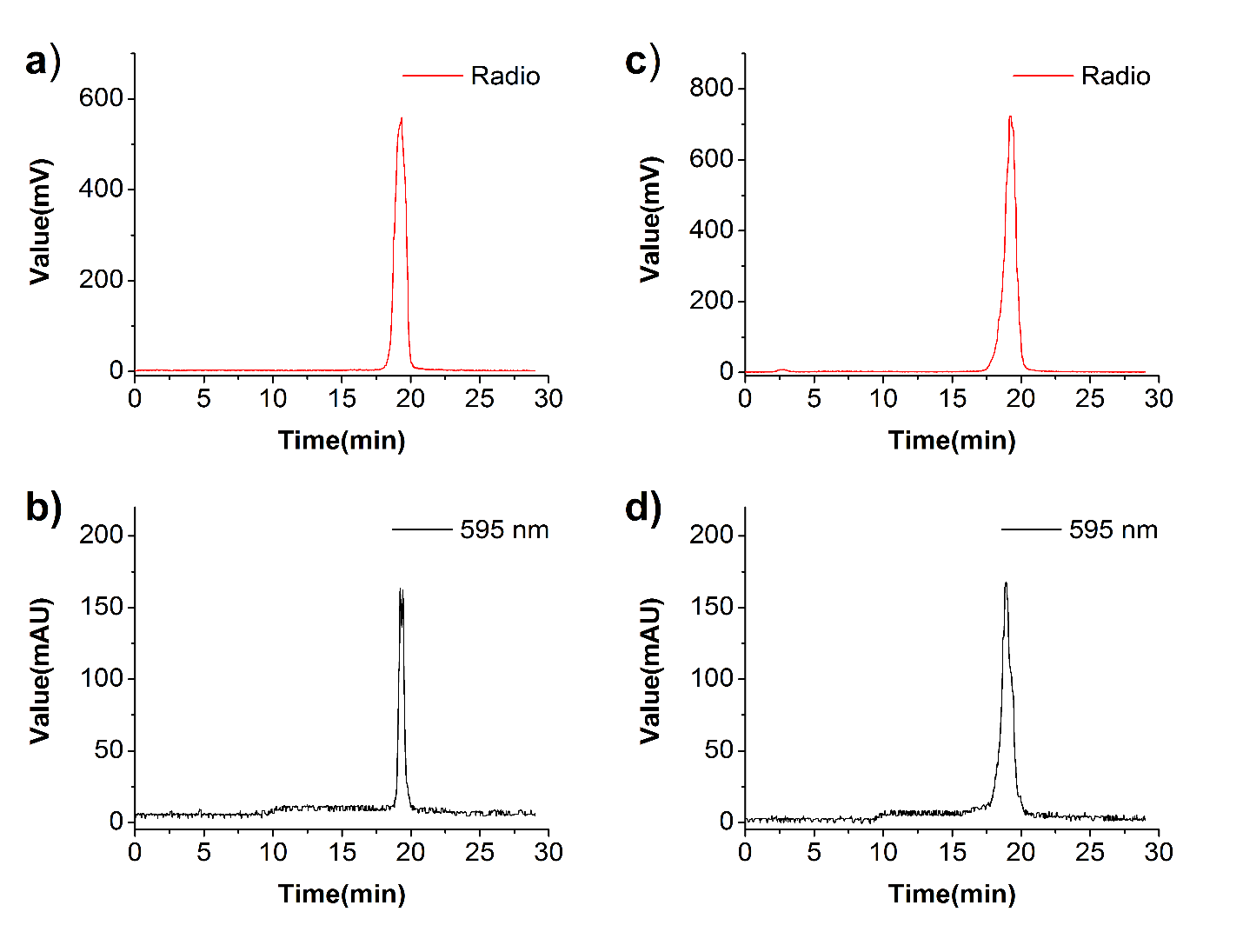


RP-HPLC spectra for ^64^Cu-BMV101. a) radioactive channel b) HPLC 595 nm UV channel c) 4h stability (in PBS) HPLC radioactive channel d) 4h stability (in PBS) HPLC 595 nm UV channel.


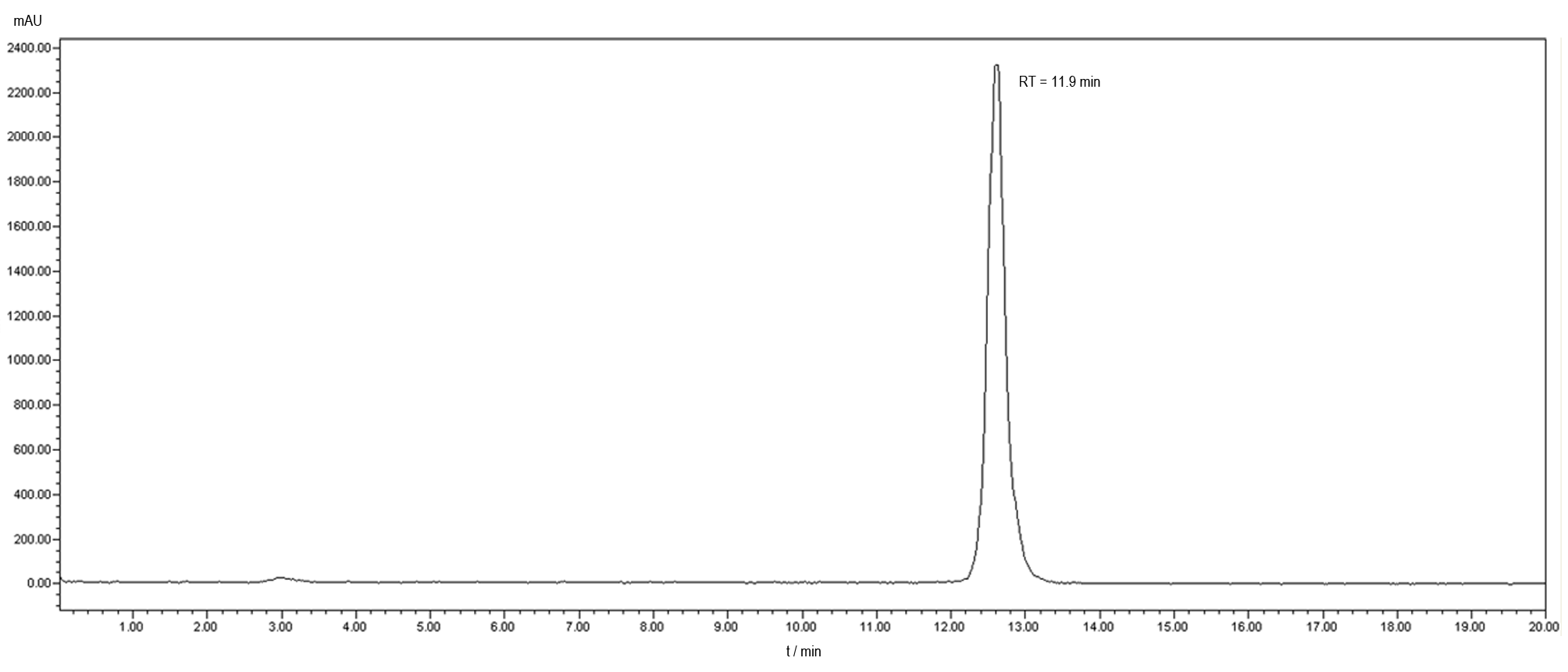


Radio-HPLC analysis of ^68^Ga-BMV101 showing the spectrum for the purified final product. The RT (retention time) for ^68^Ga-BMV101 was 11.9 min under the gradient eluent conditions outlined in the methods section.
